# Association between seated trunk control and cortical sensorimotor white matter brain changes in patients with chronic low back pain

**DOI:** 10.1371/journal.pone.0309344

**Published:** 2024-08-29

**Authors:** John R. Gilliam, Pradeep K. Sahu, Jennifer M. C. Vendemia, Sheri P. Silfies

**Affiliations:** 1 Department of Exercise Science, University of South Carolina, Columbia, SC, United States of America; 2 Department of Psychology, University of South Carolina, Columbia, SC, United States of America; 3 Physical Therapy Program, University of South Carolina, Columbia, SC, United States of America; King Khalid University, SAUDI ARABIA

## Abstract

Trunk control involves integration of sensorimotor information in the brain. Individuals with chronic low back pain (cLBP) have impaired trunk control and show differences in brain structure and function in sensorimotor areas compared with healthy controls (HC). However, the relationship between brain structure and trunk control in this group is not well understood. This cross-sectional study aimed to compare seated trunk control and sensorimotor white matter (WM) structure in people with cLBP and HC and explore relationships between WM properties and trunk control in each group. Thirty-two people with cLBP and 35 HC were tested sitting on an unstable chair to isolate trunk control; performance was measured using the 95% confidence ellipse area (CEA_95_) of center-of-pressure tracing. A WM network between cortical sensorimotor regions of interest was derived using probabilistic tractography. WM microstructure and anatomical connectivity between cortical sensorimotor regions were assessed. A mixed-model ANOVA showed that people with cLBP had worse trunk control than HC (F = 12.96; p < .001; ηp^2^ = .091). There were no differences in WM microstructure or anatomical connectivity between groups (p = 0.564 to 0.940). In the cLBP group, WM microstructure was moderately correlated (|r| = .456 to .565; p ≤ .009) with trunk control. Additionally, the cLBP group demonstrated stronger relationships between anatomical connectivity and trunk control (|r| = .377 to .618 p < .034) compared to the HC group. Unique to the cLBP group, WM connectivity between right somatosensory and left motor areas highlights the importance of interhemispheric information exchange for trunk control. Parietal areas associated with attention and spatial reference frames were also relevant to trunk control. These findings suggest that people with cLBP adopt a more cortically driven sensorimotor integration strategy for trunk control. Future research should replicate these findings and identify interventions to effectively modulate this strategy.

## 1. Introduction

Trunk control requires integrating visual, proprioceptive, and vestibular sensory information [1] and is historically recognized to involve spinal, cerebellar, and subcortical structures [2, 3]. More recent neuroimaging and electroencephalography data have unequivocally demonstrated trunk control requires the involvement of primary and secondary cortical sensorimotor brain regions [4–15]. However, the specific role of cortical sensorimotor brain structure in trunk control remains a gap in the existing literature. Previous work suggests that cortical involvement in trunk control may increase with aging due to deterioration in subcortical loops [16, 17]. Cortically driven sensory integration strategies for motor control have also been described in individuals with persistent functional limitations following musculoskeletal injury [18, 19].

Chronic low back pain (cLBP) is the leading cause of activity limitation and work absence and results in billions of dollars in healthcare expenditures [20, 21]. Individuals with cLBP consistently exhibit impairments in trunk control compared with back-healthy controls (HC) in biomechanical studies across a variety of tasks [22–30]. A separate body of neuroimaging studies indicates that cLBP is associated with alterations in cortical sensorimotor brain areas [31–34]. For example, cLBP resulted in cortical reorganization of the trunk muscles in the motor regions [35] and changes in processing proprioceptive information in somatosensory regions [36, 37]. Further, these brain changes were associated with altered movement control [38, 39]. There is a rich neuroimaging literature demonstrating brain changes through functional magnetic resonance imaging (fMRI) [30, 39–56] and voxel-based morphometry [32, 34, 48, 57–67]. However, less work has been published using diffusion magnetic resonance imaging (dMRI) to investigate differences in white matter (WM) microstructure and anatomical connectivity, which represent signaling pathways between sensorimotor brain regions. Even fewer studies have investigated the extent to which WM brain changes impact movement control in this population.

Persons with cLBP have demonstrated reduced fractional anisotropy (FA), a measure of WM microstructure, in pathways connecting cortical sensorimotor regions such as the superior longitudinal fasciculus and the corpus callosum when compared to HC [68]. Lower FA in the superior longitudinal fasciculus and corpus callosum at baseline were also associated with the development of persistent symptoms 1-year after an initial episode of back pain [69]. Increases in FA from baseline in the WM surrounding S1 have been shown to correlate with treatment response [70]. Persons with cLBP have exhibited greater anatomical connectivity (number of WM connections) between the thalamus and primary motor cortex (M1) [71] and a greater nodal degree (number of direct connections with other brain regions) of M1 than HC [72]. Only two studies in the literature have assessed the relationships between WM measures and trunk control. Early evidence indicated WM microstructure and connectivity are moderately to highly correlated with trunk control task performance [72, 73], but these studies are not without limitations. For example, both studies were performed by the same research group on similar samples. Trunk control tasks in previous studies included balancing-in-standing [73] and sit-to-stand movements [72]. These standing tasks require coordination of the trunk and lower extremities and limit the conclusions that can be drawn specific to trunk control, which is better isolated in seated paradigms that minimize the impact of lower extremity movements.

Altered trunk control has been suggested as a precursor to low back pain and a potential mechanism for the persistence of symptoms [24, 74]. Early evidence points to differences in cortical sensorimotor WM structure in this population and a link between cortical sensorimotor WM and trunk control performance, but research in this area is sparse. Exploring whether and how trunk control and cortical sensorimotor WM structure relate should provide new insights into the understanding of the neural correlates of motor performance to be exploited in the domain of rehabilitation. This cross-sectional study aimed to compare seated trunk control and sensorimotor WM structure in people with cLBP and HC and explore relationships between WM properties and trunk control in each group. We hypothesized that people with cLBP would demonstrate poorer seated trunk control, reduced cortical sensorimotor WM microstructural integrity, and increased anatomical connectivity between cortical sensorimotor regions. We hypothesized that reduced WM microstructural integrity and increased anatomical connectivity would be associated with poorer trunk control task performance.

## 2. Materials and methods

### 2.1 Participants

A sample of 32 participants with cLBP and 35 demographically similar [age (±5 years), sex, and BMI (±2 kg/m^2^) matched] HC were recruited from the local community (Table 1). Participants represent a subset from a larger study (R01-HD095959) that additionally underwent biomechanical testing of trunk control. The low back region was defined as the area below the 12^th^ ribs and above the gluteal folds. Participants who experienced lower extremity neurological symptoms related to back pain were excluded. Participants with cLBP were included if: 1) they had symptoms for >3 months; 2) experienced symptoms at least half the days in the past 3–6 months; and 3) back pain limited their ability to perform daily activities [75]. Detailed inclusion and exclusion criteria for both groups can be found in the supporting information S1 Appendix. All participants gave their written informed consent prior to testing. The study was approved by the Institutional Review Board of the University of South Carolina (Pro00079198) and was conducted according to the principles expressed in the Declaration of Helsinki.

**Table 1:** Participant demographics.

	cLBP (n = 32)		HC (n = 35)		p-value
Sex	21F		24F		0.78
# seeking treatment	26		NA		
	Mean	SD	Mean	SD	
Age	29.3	11.0	29.2	11.0	0.99
Body Mass Index (BMI)	26.2	3.9	25.1	3.1	0.23
RTF Impact (9–50)	19.2	5.5	9.3	0.7	< .01
Pain: 7-day avg (0–10)	4.4	1.4	NA		
Oswestry Disability Index (0–50)	8.1	3.7	NA		
Tampa Scale of Kinesiophobia-17 (17–68)	33.6	6.1	NA		
CPAQ (0–120)	81.9	15.2	NA		
CES-D (0–60)	14.2	12.1	6.9	5.6	< .01
PCS (0–52)	9.5	8.9	2.2	3.5	< .01
STAI-State Anxiety (20–80)	46.2	5.7	42.3	4.6	< .01
STAI-Trait Anxiety (20–80)	39.9	12.5	30.9	8.7	< .01
Modified Baecke PA (0–15)	9.6	1.8	9.2	1.8	0.44

RTF: NIH Task Force on Research Standards for Chronic Low Back Pain; CES-D: Center for Epidemiologic Studies Depression Scale (CES-D); PCS: Pain Catastrophizing Scale; PA: Physical Activity; CPAQ: Chronic Pain Acceptance Questionnaire; STAI: State and Trait Anxiety Inventory

### 2.2 Questionnaires and clinical tests

Before testing, the pain intensity of all participants over the past week was evaluated by an 11-point numeric pain rating scale [76]. Habitual physical activity was assessed in all participants using the Modified Baecke Physical Activity Questionaire [77]. We also collected additional self-report questionnaires to characterize the sample of individuals with cLBP in this study. Low back-specific function was assessed using the Oswestry Disability Index [78]. Pain impact was assessed as a part of the NIH Research Task Force Minimal Data Set [75]. We measured pain acceptance and catastrophization using the Chronic Pain Acceptance Questionaire [79] and the Pain Catastrophization Scale [80], respectively. Fear of movement was assessed using the 17-item Tampa Scale of Kinesiophobia [81]. Depression was measured using the Center for Epidemiological Studies-Depression Scale (CES-D) [82]. State and trait anxiety were evaluated with the State-Trait Anxiety Inventory (STAI) [83]. These self-report measures have been validated for individuals with chronic low back pain [84–87].

### 2.3 Seated trunk control

#### 2.3.1 Procedure

**Fig 1:**
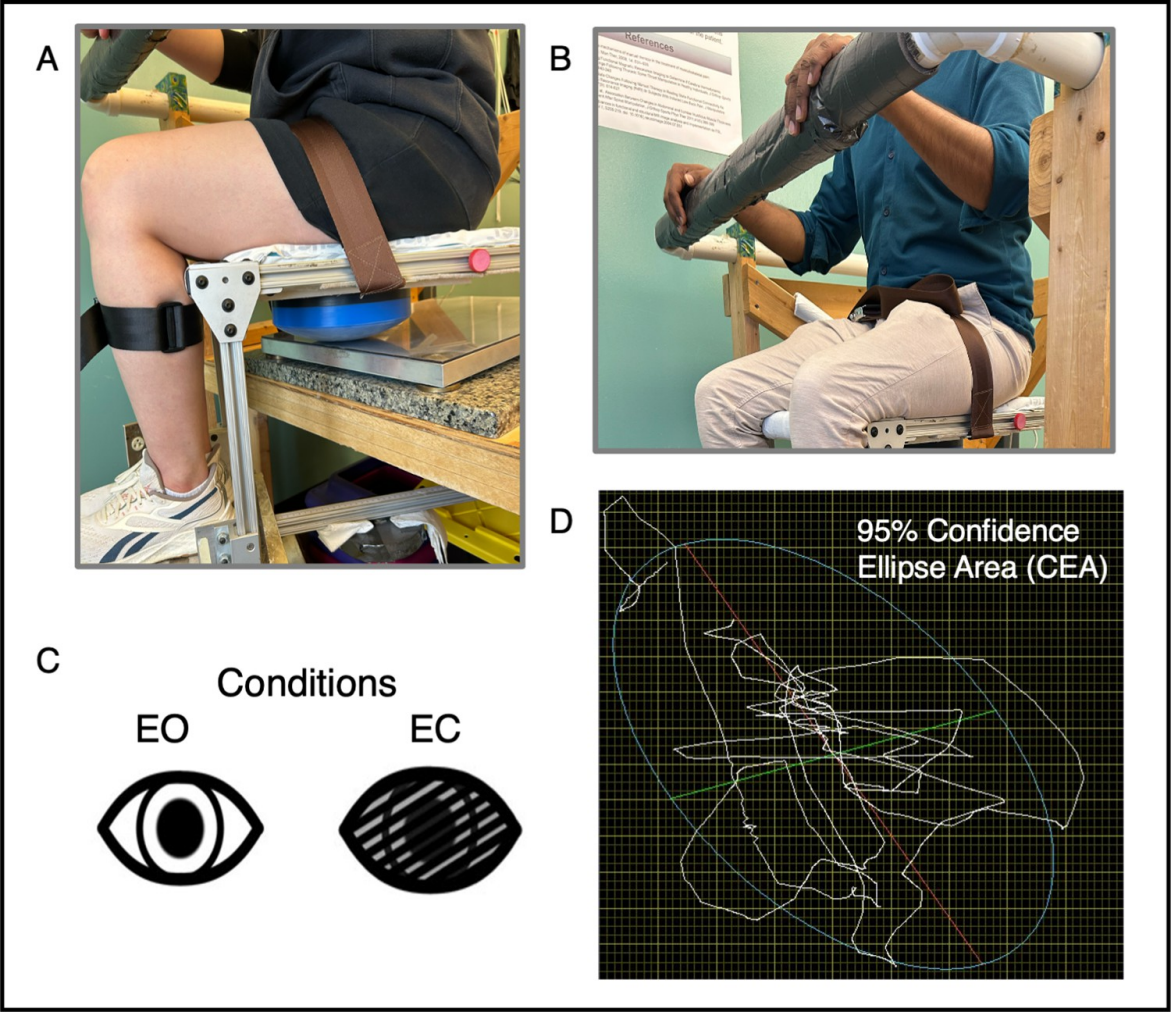
Seated trunk control task. **A.** An unstable chair, developed to isolate trunk control, positioned atop a force plate. An adjustable hemisphere mounted under the chair makes sitting unstable and allows for standardization of position across different thigh lengths. The hips, knees, and ankles are positioned in 90 degrees of flexion, and straps are placed around the legs to minimize movement of the lower extremities. **B.** A safety bar positioned anteriorly allows participants to stabilize and rest the trunk muscles between trials. **C.** Two conditions were tested: eyes open (EO) and eyes closed (EC). **D.** 95% confidence ellipse area (CEA) around a single participant’s center-of-pressure (COP) tracing. 95% CEA represents how much a participant deviated from their mean COP.

A seated testing apparatus designed to isolate trunk motor control by minimizing the contribution of the lower extremities was used for trunk control testing [22]. Participants sat on an unstable chair, including a seat and adjustable footrest, with a solid hemisphere mounted under the seat (Fig 1A). This setup creates an unstable surface and was selected for this investigation because it requires active trunk motor control to maintain upright sitting. The chair is balanced without a participant seated and is adjustable to allow the position of the hemisphere and starting position to be standardized. Participants sat in a neutral spine position with their hips, knees, and ankles flexed at 90°. Seated trunk control was measured using a force plate (Kistler, Novi, MI, USA) under the chair. During testing, participants were asked to sit with their arms crossed across their chest, with each hand touching the contralateral clavicle.

A protocol was developed through pilot testing that stabilized task performance with practice and determined the appropriate number of practice trials and rest breaks. Participants were provided real-time visual feedback of their center of pressure (COP) during initial practice trials of multidirectional movement to allow familiarization with the chair and how trunk movement influenced COP changes. Following practice, the visual feedback of COP was removed for trunk control testing. Participants were instructed to “balance yourself and remain as still possible” and control upright sitting for 30 seconds. Three trials with eyes open (EO) followed by three trials with eyes closed (EC) (Fig 1C) were recorded following a 30-second practice trial in each condition. This number of trials was selected to avoid excessive trunk muscle fatigue. A 30-second rest break was provided between each trial. During rest, participants steadied themselves by holding on to a safety bar located in front of them to prevent the accumulation of trunk muscle fatigue (Fig 1B).

#### 2.3.2 Data processing

Time series COP data was collected at 2,400 Hz, filtered using a piecewise linear filter, and down-sampled to 240 Hz using custom software (Labview, version 8.6; National Instruments, Austin, TX, USA). To quantify seated trunk control performance, we calculated a 95% confidence ellipse area (CEA_95,_ mm^2^) of the COP (Fig 1D) during EO and EC conditions and averaged across three trials. Mean CEA_95_ represents the area that contained 95% of the COP tracing during testing. A higher value indicates greater deviation from the task instructions and, therefore, worse performance.

### 2.4 Diffusion MRI

#### 2.4.1 MRI acquisition

Magnetic resonance data were acquired at the McCausland Center for Brain Imaging on a 3 Tesla Siemens Prisma scanner (Erlangen, Germany) equipped with a 16-channel head coil. A three-dimensional sagittal T1-weighted magnetization-prepared rapid acquisition with gradient echo (MPRAGE) sequence (Repetition Time (TR): 2250 ms, Echo Time (TE): 4.11 ms, voxel size: 1 mm isotropic, 192 axial slices covering the whole brain; matrix = 256x256) was used to acquire high-resolution structural images. Diffusion data were acquired using an echo-planar imaging (EPI) sequence (TR: 2945 ms, TE: 104 ms, matrix = 128x128, voxel size: 2.2 mm isotropic, 57 slices) with two gradients of diffusion weighting (b = 1000 s/mm^2^, b = 2000 s/mm^2^) acquired in 137 directions.

#### 2.4.2 Parcellation of gray-matter regions of interest

**Fig 2:**
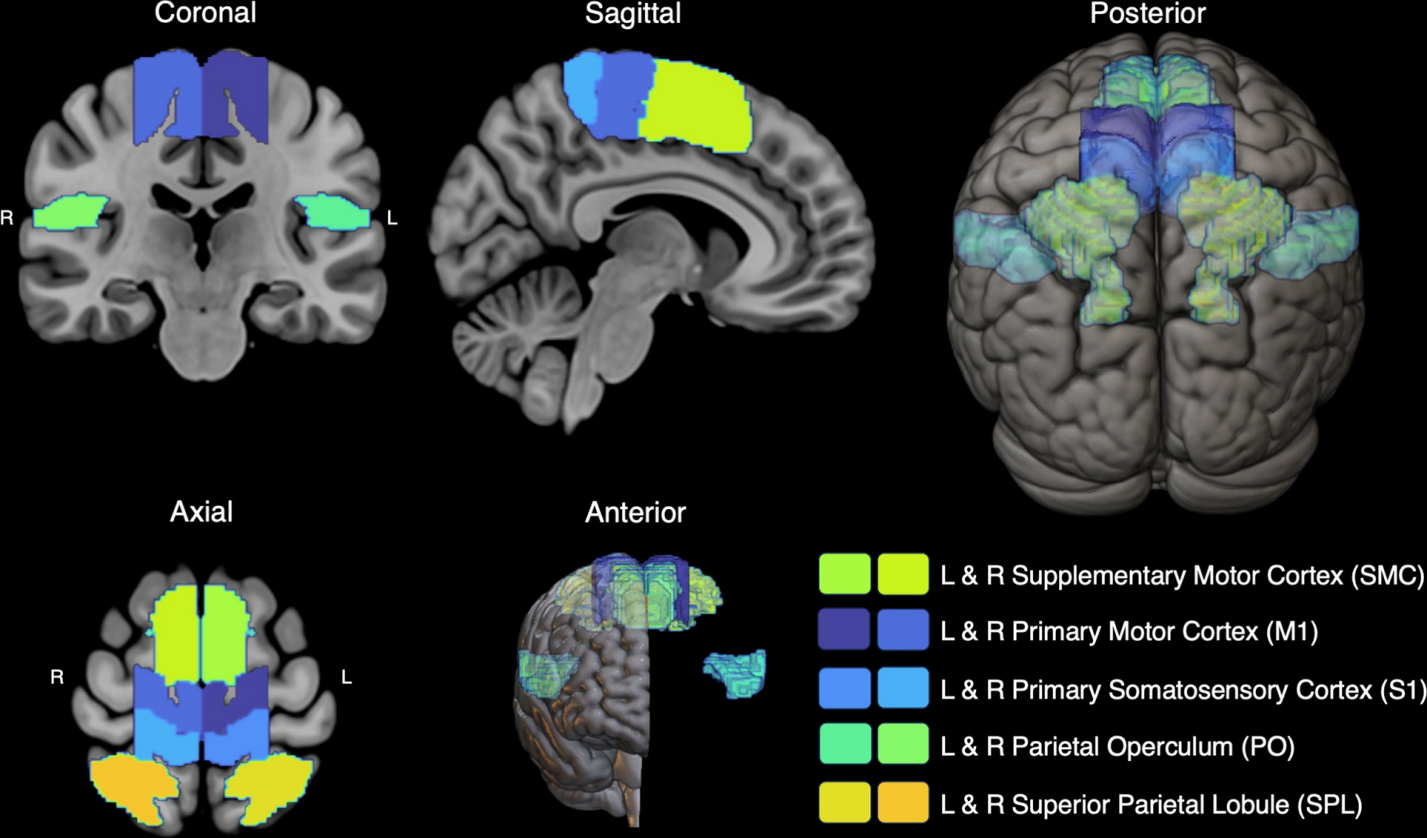
Cortical Sensorimotor Gray Matter Atlas for the Trunk (CSMGA-T). The CSGA contains primary and secondary/supplementary sensorimotor brain regions of interest in both the left and right hemispheres, including the supplementary motor cortex, primary motor and somatosensory cortices, parietal operculum, and superior parietal lobule. The medial primary motor and somatosensory cortices were selected to focus our investigation on the gray matter considered most relevant to the trunk.

In order to study sensorimotor gray-matter regions specific to trunk control, parcellations were developed using a standardized method previously described [88]. Briefly, we selected common brain atlases, including the Automated Anatomical Labeling (AAL [89]), Desikan [90], Destrieux [91], Glasser [92], Hammersmith [93], Harvard-Oxford [94], Human Motor Area Template (HMAT [95]), and Juelich [96] atlases. Dice Coefficients were calculated as a measure of similarity between atlases and to determine which sensorimotor regions from each atlas would be carried forward. We then performed an adjusted mutual information analysis for each region of interest, comprised of individual regions from pertinent atlases. Adjusted mutual information results were then used to inform a weighted multi-atlas voting algorithm, where individual regions that accounted for more mutual information were more heavily weighted. Five bilateral primary and secondary cortical sensorimotor regions of interest (ROIs) were selected a priori, including the supplementary motor cortex (SMC), the primary motor (M1) and somatosensory (S1) cortices, the parietal operculum (PO), and the superior parietal lobule (SPL). The bilateral M1 and S1 were further refined to include only the medial portion associated with the trunk in previous literature [37, 70, 97, 98]. The cortical sensorimotor gray matter atlas for the trunk is presented in Fig 2. A bilateral network is particularly relevant to the trunk because of the nature of trunk control and the reliance on integrating information like changes in muscle length and joint position into motor outputs on both sides of the body. Registration of cortical sensorimotor ROIs between standard (MNI152 T1 2-mm brain) and diffusion images was carried out using FSL’s (FMRIB Software Library, https://www.fmrib.ox.ac.uk/fsl) FLIRT and FNIRT [99]. Registration matrices for diffusion to T1 images and T1 to standard images were concatenated, and a single registration was performed. Each registered ROI was visually inspected for fit for each participant.

#### 2.4.3 dMRI processing

Diffusion data processing and analysis were performed using FMRIB’s Diffusion Toolbox [100]. Raw diffusion images underwent FSL’s topup and eddy correction for susceptibility and eddy current-induced distortions, respectively. Eddy correction was performed with FSL’s “repol” option for outlier replacement, and eddy-adjusted bvecs were used for subsequent analytical steps [101]. Skull-stripping was performed using FSL’s brain extraction tool to remove non-brain tissue, and a diffusion tensor model was fit at each voxel to determine voxel-wise Fractional Anisotropy (FA), Mean Diffusivity (MD), and eigenvalues of diffusion.

Four key WM microstructure metrics derived from dMRI data are FA, MD, Axial Diffusivity (AD), and Radial Diffusivity (RD). FA measures the directionality of water diffusion in tissue. It reflects the degree of anisotropy (directional dependence) of water diffusion, providing insight into the alignment of fibers within a voxel. FA values range from 0 (isotropic diffusion) to 1 (highly anisotropic diffusion). FA indicates fiber density, axonal diameter, and myelination in WM. MD represents the average rate of water diffusion within a voxel and measures the overall mobility of water molecules. It is calculated as the mean of the three eigenvalues of the diffusion tensor, which represents diffusion in three orthogonal directions. Higher MD values may indicate increased extracellular space, as observed in edematous or degenerative tissue. AD measures the rate of diffusion along the principal axis of the diffusion tensor. It is equal to the largest eigenvalue of the diffusion tensor. AD is thought to be sensitive to axonal integrity, and changes in AD are often associated with axonal injury or loss. RD quantifies the rate of water diffusion perpendicular to the principal diffusion axis and is the average of the second and third eigenvalues. RD is sensitive to changes in myelination, and increased RD is often associated with demyelination processes [102–107].

#### 2.4.4 Tractography

**Fig 3:**
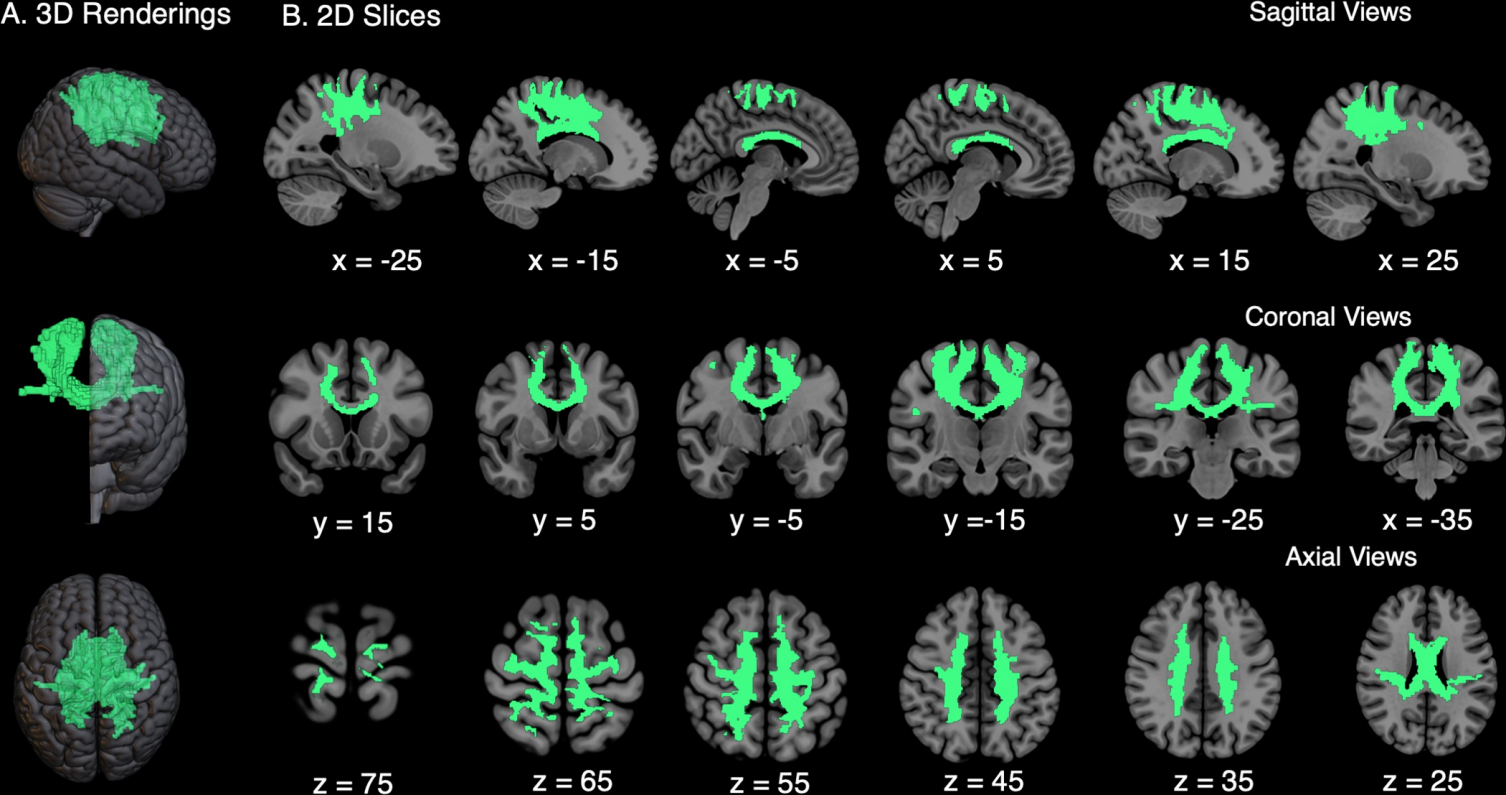
Cortical Sensorimotor White Matter Tracts for the Trunk (CSMWT-T). **A.** The CSMWT-T contains contiguous 3-dimensional tracts connecting 5 different cortical sensorimotor gray matter areas bilaterally, including the supplementary motor cortex, primary motor and somatosensory cortices, parietal operculum, and superior parietal lobule. **B.** The 2-dimensional representation of these tracts in the sagittal, coronal, and axial views.

An ROI-to-ROI probabilistic tractography was applied in diffusion space to reconstruct WM pathways between our 10 cortical sensorimotor ROIs for each participant. Tractography was estimated using Bayesian Estimation of Diffusion Parameters Obtained using Sampling Techniques (BEDPOSTX) [108] to build default distributions of diffusion parameters at each voxel. Tractography was applied using FSL’s Diffusion Toolbox and probtrackX2 [109] (parameters: 5000 individual pathways drawn through the probability distributions on principle fiber direction, curvature threshold set at 0.2, 200 maximum steps, step length of 0.5 mm, and distance correction). White-matter maps were created for each participant from their high-resolution T1-weighted image. White-matter maps were normalized to diffusion space, visually inspected for fit, and used as a waypoint mask. An exclusion mask was hand-drawn for each participant just below the corpus callosum and PO to restrict tractography to the cortical sensorimotor tracts. White matter pathways were thresholded at a 6% maximum. This threshold was determined based on visual inspection of results, where higher threshold values excluded pathways connecting the PO. Cortical sensorimotor WM tracts for a representative participant can be visualized in Fig 3.

#### 2.4.5 Cortical sensorimotor WM microstructure

Our primary objectives were to compare cortical sensorimotor WM tract volumes, FA, MD, RD, and AD between groups and to analyze the relationship between these measures and seated trunk control task performance (CEA_95_). Thresholded and binarized cortical sensorimotor WM tracts for each participant were multiplied by FA, MD, RD, and AD images generated from fitting the diffusion tensor model. Mean FA, MD, RD, and AD values were calculated for each participant within their reconstructed WM tracts (Fig 3).

#### 2.4.6 Pairwise anatomical connectivity

In addition to our primary analyses, we assessed pairwise anatomical connectivity between our cortical sensorimotor ROIs. This allows us to identify which connections within our cortical sensorimotor WM tracts are most relevant to group status and task performance. Anatomical connectivity between ROIs was defined as the number of streamlines arriving in one ROI when another ROI was seeded and vice versa. The anatomical connectivity between regions A and B was defined as the number of probabilistic streamlines arriving at region B when region A was seeded, averaged with the number of probabilistic streamlines arriving at region A when region B was seeded. The calculation of streamlines was corrected based on the distance traveled by the streamline connecting regions A and B. To control for the unequal size of gray-matter regions, the number of streamlines connecting each pair of regions was divided by the total sampling number from the seeded region reaching all ROIs in the network.

### 2.5 Statistical analysis

Statistical analyses were conducted in SPSS, version 29 (IBM, Armonk NY), and MATLAB, version 2022b (MathWorks, Natick MA). Alpha was set at 0.05. Demographic and clinical data were assessed for between-group differences with independent t-tests, and Mann-Whitney U tests for continuous variables. Chi-square (χ^2^) tests were used for categorical variables. Where baseline group differences in clinical data existed, we assessed correlations between clinical measures and CEA_95_. Correlations were interpreted as negligible (0.0–0.10), weak (0.11–0.39), moderate (0.40–0.69), strong (0.70–0.89), and very strong (0.90–1.0) [110]. A mixed-model analysis of variance (ANOVA) was used to compare trunk control performance between groups and across conditions. Group (cLBP vs. HC) and condition (EO vs. EC) were considered fixed factors. Partial eta squared (ηp2) was calculated to determine the effect size. Between-group differences for tract volume, FA, MD, RD, AD, and pairwise anatomical connectivity values were assessed using Mann-Whitney U tests. Spearman correlations were calculated to determine the relationships between measures of microstructure, pairwise anatomical connectivity, and CEA_95_ in each group. We employed permutation threshold testing to determine the significance of correlations. We created an empirical distribution of null hypothesis values with 10,000 permutations of our data. By comparing the observed test statistic to the empirical distribution, we can calculate p-values that reflect the likelihood of obtaining the observed results under the null hypothesis. This method controls for multiple comparisons and reduces the likelihood of type I errors, providing a robust measure [111, 112].

## 3 Results

### 3.1 Demographic and clinical data

No between-group differences were observed for sex, age, or BMI. Group differences were observed for measures of depression, pain catastrophizing, and state and trait anxiety, where the cLBP group demonstrated higher scores on these measures (Table 1). There were no associations between depression, pain catastrophizing, or anxiety and CEA_95_ (p>0.05). No group differences were observed for physical activity.

### 3.2 Seated trunk control

**Fig 4:**
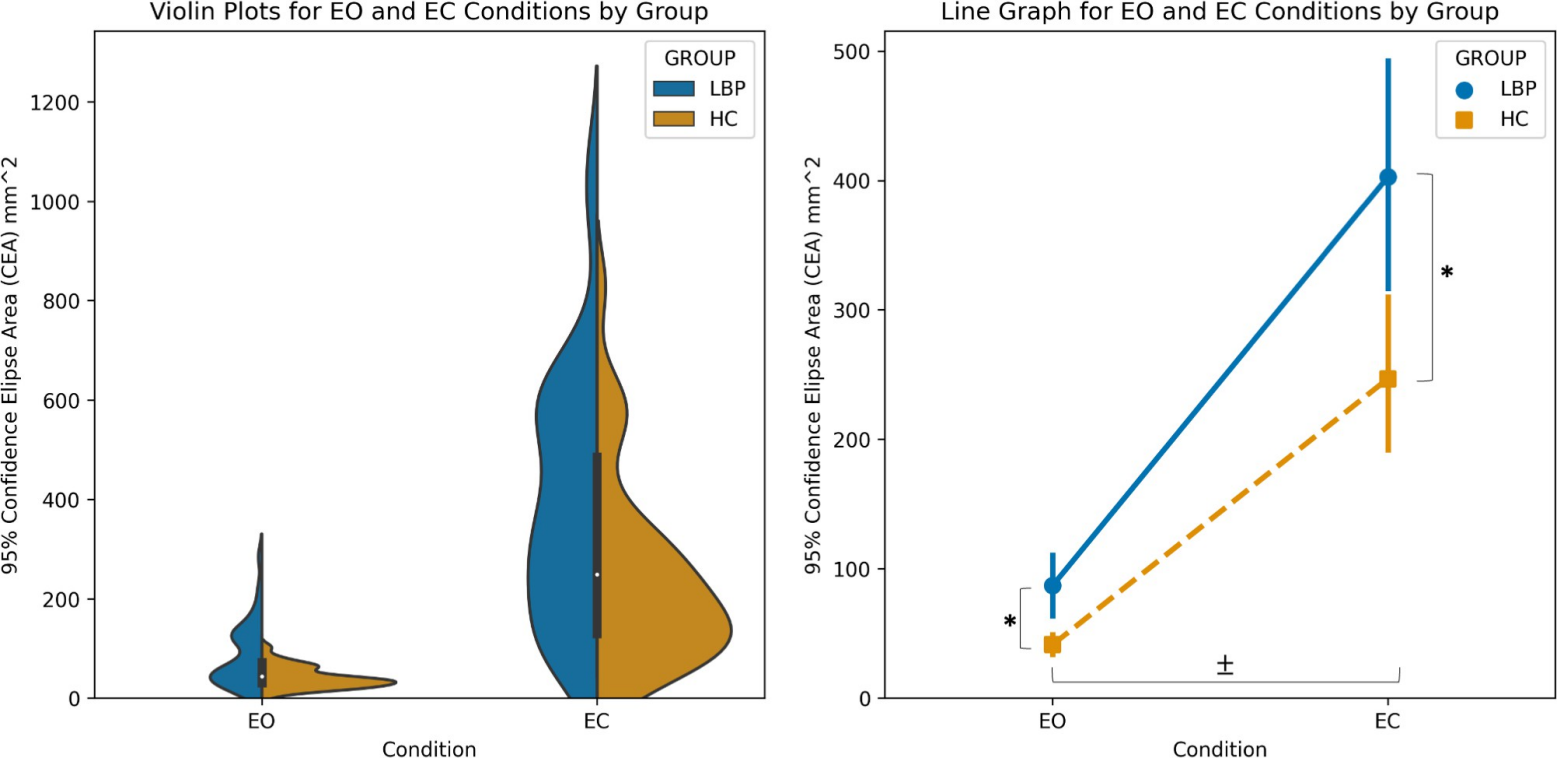
Seated trunk control performance by group and condition. **Left:** Violin plots displaying the distribution of seated trunk control performance by condition for each group. The chronic low back pain group (cLBP) presents a bimodal distribution for both tasks. Both groups exhibit skewness towards poor performance. **Right:** Line plots representing group means and 95% confidence intervals for each condition. ANOVA reveals main effects for group (*; p < .001) and condition (±; p < .001), where the cLBP group performed more poorly than healthy controls, and both groups performed more poorly with eyes closed. A trend (p = .051) for a group x condition interaction was observed where the change in performance between eyes open and eyes closed was larger in the cLBP group.

Plots displaying the distribution of trunk control performance for each group and conditions are presented in Fig 4, left. Mixed-model ANOVA revealed main effects for group (F = 12.96; p < .001; ηp2 = .091) and condition (F = 86.56; p < .001; ηp2 = .400). The cLBP group performed worse than HC for both conditions, and both groups performed worse with EC (Fig 4, Right). A trend for a group x condition interaction (F = 3.87; p = .051; ηp2 = .029) was observed where the change in performance between EO and EC was larger in the cLBP group.

### 3.3 Cortical sensorimotor WM microstructure

**Fig 5:**
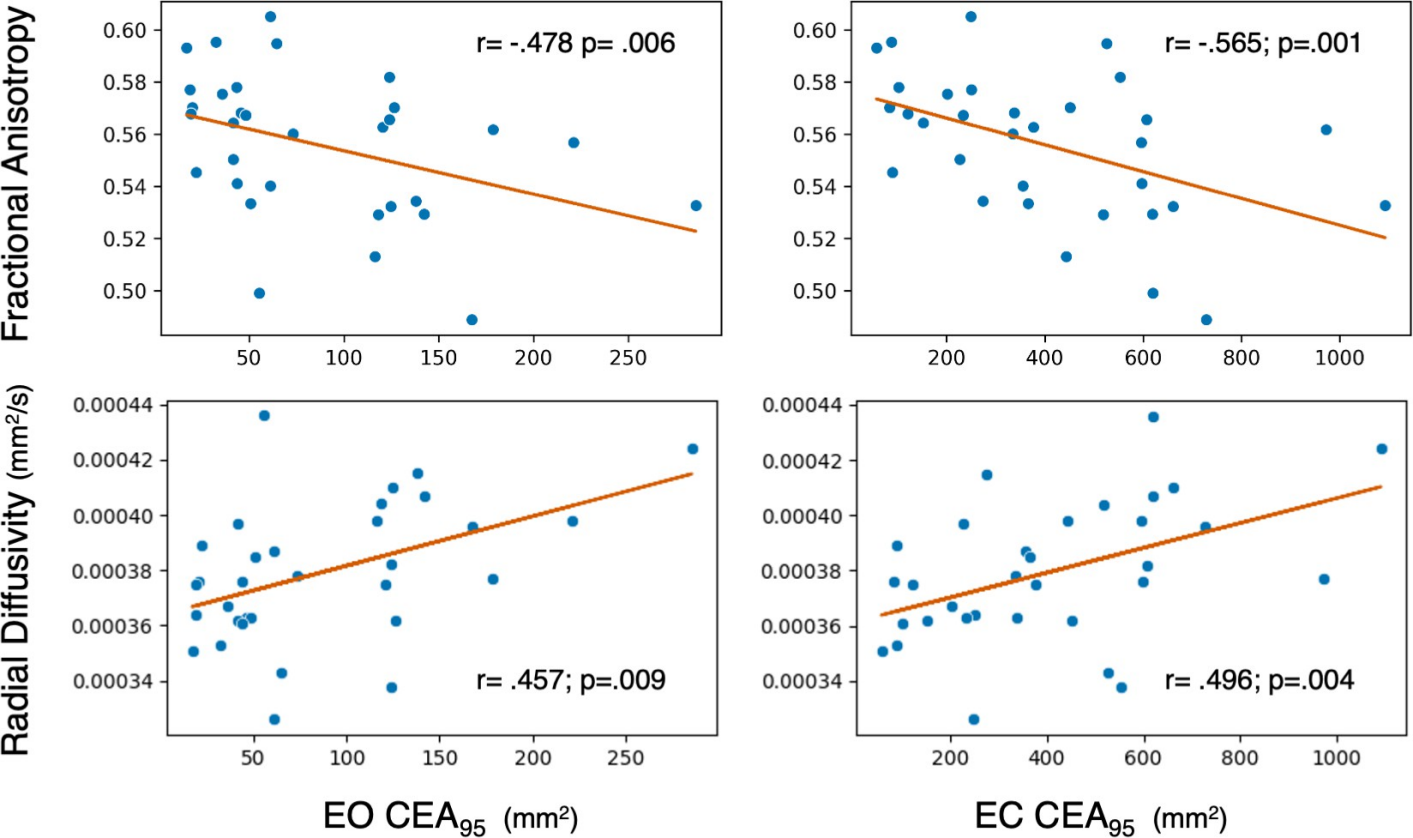
Scatter plots of cortical sensorimotor white matter microstructure and seated trunk control in chronic low back pain. **Top Left:** Scatter plot of cortical sensorimotor tract fractional anisotropy (FA) and 95% Confidence Ellipse Area (CEA) for the eyes open (EO) condition. **Top Right:** Scatter plot of cortical sensorimotor tract FA and 95% CEA for the eyes closed (EC) condition. **Bottom Left:** Scatter plot of cortical sensorimotor tract radial diffusivity (RD) and 95% Confidence Ellipse Area (CEA) for the EO condition. **Bottom Right:** Scatter plot of cortical sensorimotor tract RD and 95% CEA for the EC condition.

There were no between-group differences for cortical sensorimotor WM microstructure for any measure (p = 0.564–0.940; S1 Table). The cLBP group exhibited negative correlations between FA and seated trunk control for the EO (r = -.478; p = .006) and EC (r = -.565; p = .001) conditions (Fig 5, Top). Correlations also reveal a positive association between RD and trunk control in the EO (r = .457; p = .009) and EC (r = .496; p = .004) conditions (Fig 5, bottom). Tract volume (p = .075-.153), MD (p = .193-.314), and AD (p = .277-.798) were not associated with seated trunk control in the cLBP group. We found no relationships between cortical sensorimotor WM microstructure and seated trunk control in the HC group (p = .244-.895). Therefore, cortical sensorimotor WM microstructure was associated with trunk control in the cLBP group but not in HC.

### 3.4 Pairwise anatomical connectivity

**Fig 6:**
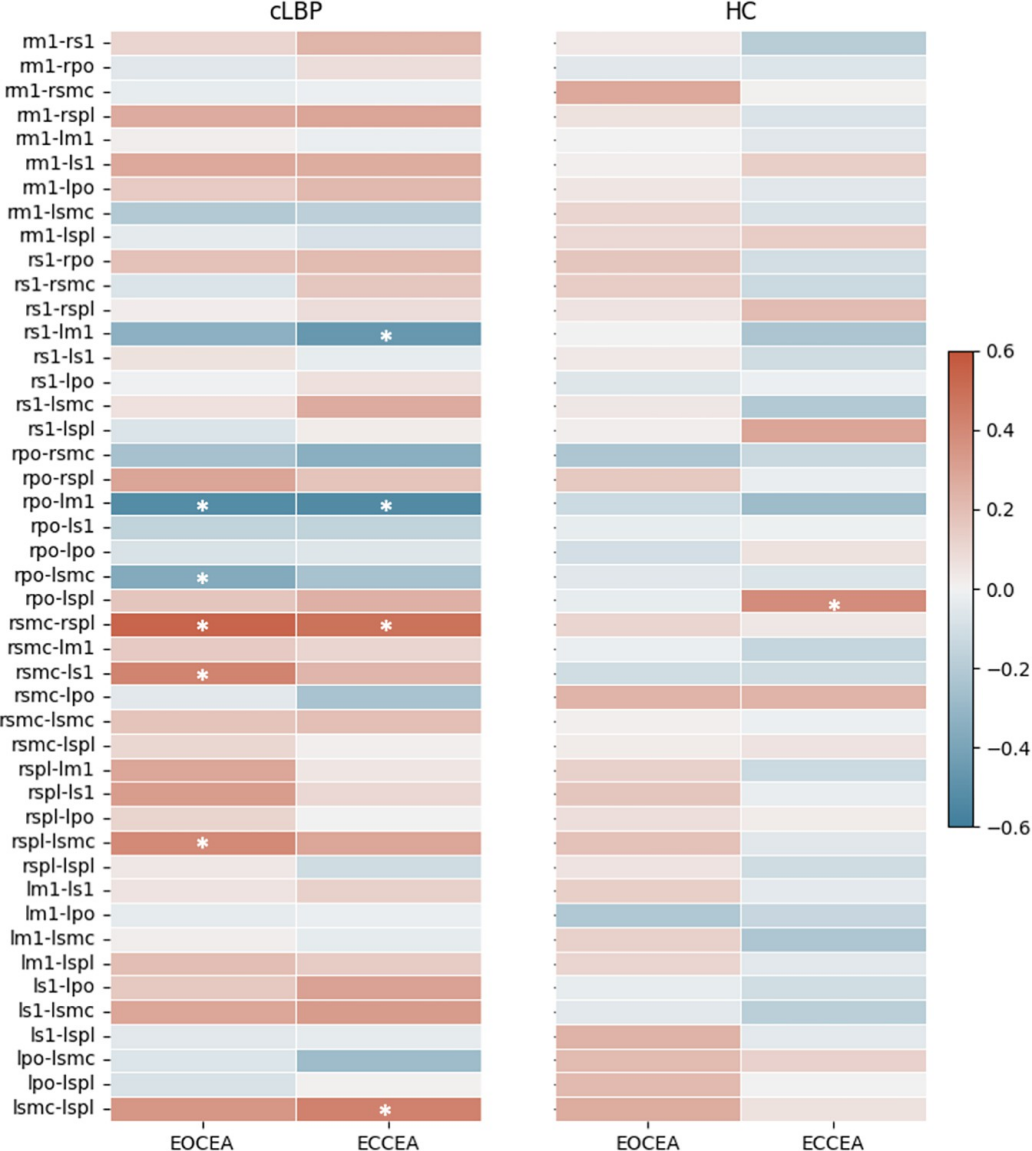
Correlation heat maps for pairwise anatomical connectivity between cortical sensorimotor regions of interest and seated trunk control. **Left:** Correlation heat map for chronic low back pain participants (n = 32). **Right:** Correlation heat map for healthy control participants (n = 35). *Asterisks indicate significant correlations (p<0.05) after strict permutation threshold testing.

We observed no group differences in pairwise anatomical connectivity for any region pair (S2 Table). In the cLBP group, five region pairs were associated with trunk control performance in the EO condition, and 4 region pairs were associated with performance in the EC condition. The HC group had only a single region pair (RPO-LSPL; r = .381 p = .024) that was positively associated with trunk control performance in the EC condition (Fig 6), suggesting that cortical sensorimotor anatomical connectivity in the cLBP participants is more strongly associated with trunk control performance.

**Fig 7:**
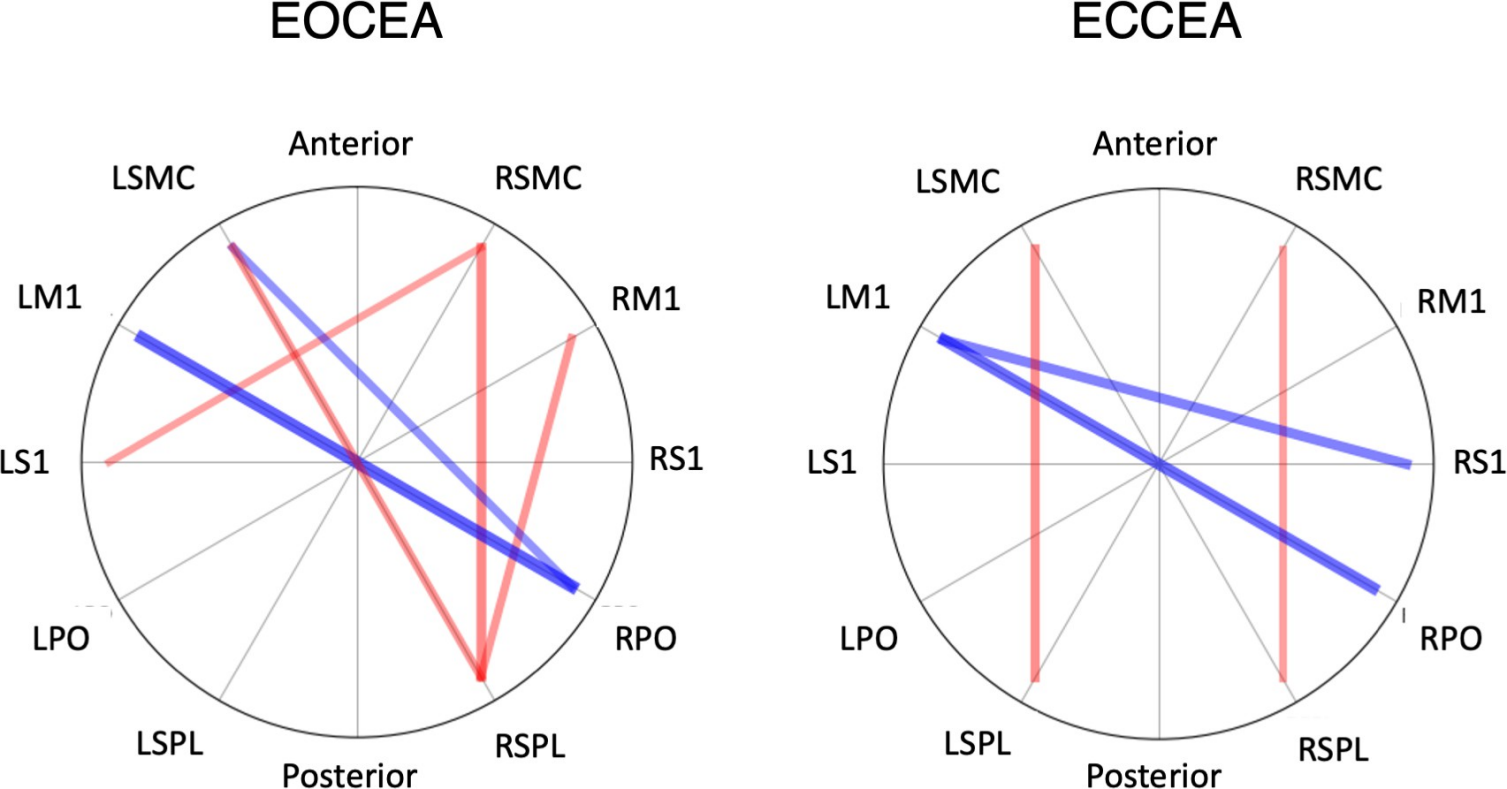
Chord diagrams of correlations between pairwise anatomical connectivity between cortical sensorimotor regions of interest and seated trunk control in chronic low back pain participants. Significant correlations (p<0.05) after strict permutation threshold testing are presented where negative correlations are blue and positive correlations are red. The strength of a given relationship is represented by the width and intensity of the line. **Left:** Correlation values for 95% confidence ellipse area (CEA) in eyes open (EO) condition. **Right:** Correlation values for 95% CEA in eyes closed (EC) condition.

For the cLBP group, anatomical connectivity between RPO-LM1 was negatively associated with both EO (r = -.618; p = .002) and EC (r = -.569; p = .001) CEA_95_. Anatomical connectivity between the RSPL-RSMC was positively correlated with task performance under both EO (r = .468; p = .002) and EC (r = .377; p = .006) conditions. Other region pairs associated with trunk control for people with cLBP in the EO condition include RPO-LSMC (r = -.419; p = .034), RSPL-LSMC (r = .408; p = .025), and LS1_RSMC (r = -.569; p = .030) (Fig 7, Left). Region pairs only associated with performance under the EC condition in persons with cLBP were RS1-LM1 (r = -.491; p = .007), and LSPL-LSMC (r = .442; p = .018) (Fig 7, Right).

## 4 Discussion

To the authors’ knowledge, this is the first study to use dMRI to provide insight into the relationships between white matter structure and seated trunk control in cLBP. We compared seated trunk control and cortical sensorimotor WM between persons with cLBP and matched HC. In line with our hypothesis, persons with cLBP demonstrated poorer seated trunk control. However, counter to our expectations, we did not observe differences between groups in cortical sensorimotor white matter microstructural integrity or anatomical connectivity. We also hypothesized that white matter microstructure and anatomical connectivity would be associated with poorer trunk control task performance, which was only the case for the cLBP group.

The participants with cLBP in this study had moderate pain scores. They also exhibited relatively low disability due to back pain. This relationship may be influenced by relatively high scores on the Chronic Pain Acceptance Questionnaire, which evaluates activity engagement and pain willingness [79]. Roughly one-third (12/32) of participants with cLBP exceeded 37 on the Tampa Scale of Kinesiophobia, placing these individuals at a higher risk for poorer outcomes [81]. The cLBP group had higher scores for self-report measures of depression, pain catastrophizing, and state and trait anxiety compared with HC, but group means remained below clinical cutoff scores, and these cognitive-behavioral measures were not related to trunk control. The groups were not different for physical activity level, which may contribute to the overlap of distributions between groups for trunk control performance.

We found the cLBP group demonstrated poorer seated trunk control performance compared with HC. This aligns with previous evidence of trunk control impairment in this population [22–30, 113]. More specifically, these findings replicate previous studies using similar testing methods in individuals with low back pain [22, 113]. Radebold et al. [113] compared trunk control performance between cLBP and healthy controls using an unstable chair and conditions with eyes open and eyes closed. Individuals with cLBP demonstrated poorer trunk control, especially under more challenging conditions [113]. Sung et al. [22] used an identical testing protocol to ours in persons with sub-acute back pain, except that trunk control tasks were performed for 60 instead of 30 seconds [22]. These authors reported large effect sizes between groups where our between-group effects were moderate and reported a group x time interaction where we observed a trend. This may be due to the acuity of symptoms, the longer duration of trials, or that the previous study required clinical evidence of motor control impairments as inclusion criteria. The authors discussed the persistence of trunk control impairment being an important factor in the lack of symptom resolution from the acute to subacute phase [22]. Here, we provide additional evidence that seated trunk control impairment persists past the sub-acute into the chronic phase and should be considered a relevant factor in the persistence of symptoms.

We used tractography to reconstruct cortical sensorimotor white matter tracts specific to the trunk and compare the white matter microstructure within these tracts between individuals with cLBP and HC. We did not detect differences between groups. The literature in this area is mixed, and no two studies have implemented the same processing and analytical methods. In the single study that reported differences between cLBP and HC, Ma et al. [68] assessed 50 white matter regions from the Johns Hopkins University (JHU) ICBM-DTI-81 white matter atlas and found differences in fractional anisotropy in the anterior and posterior thalamic radiations, the corpus callosum, the anterior corona radiata, and the superior longitudinal fasciculus between cLBP (n = 24) and HC (n = 22) groups [68]. Worth mentioning, in the study by Ma et al. [68], significant between-group differences in age were reported (cLBP 15 years older on average), and age-related changes in white matter microstructure have been reported in the literature [114]. Alternatively, and in agreement with the findings in the present study, Pijnenburg and colleagues failed to find group differences in fractional anisotropy, mean diffusivity, radial diffusivity, or axial diffusivity using regions selected for their role in sensory or motor processing [73]. Similarly, Mao et al., compared white matter microstructure of the thalamo-motor and thalamo-somatosensory fibers between 54 individuals with cLBP and 54 HC and found no differences in fractional anisotropy or mean diffusivity in these pathways [71].

In the cLBP group, fractional anisotropy and radial diffusivity of cortical sensorimotor white matter tracts were correlated to trunk control in both the eyes open and eyes closed conditions (Fig 5). The increased task difficulty of the eyes closed condition, absent visual feedback, amplified the ability to detect this relationship. We found moderate negative correlations explaining 22–32% of the variance between fractional anisotropy and task performance, indicating that lower fractional anisotropy was associated with worse trunk control. We also found that radial diffusivity had moderate positive correlations (20–24% of the variance) with task performance, meaning that higher radial diffusivity was related with poorer trunk control. Taken together, white matter microstructure findings suggest that a reduction in myelination of cortical sensorimotor tracts results in impaired trunk control [102–107]. We did not observe relationships between mean diffusivity or axial diffusivity and trunk control, suggesting these measures may be less sensitive or that trunk control performance is not related to changes in the extracellular space, degeneration, axonal injury, or loss. In a cLBP cohort (n = 18), Pijnenburg et al. [73] reported negative (r = -.73) correlations between fractional anisotropy in the superior cerebellar peduncle and proprioceptive weighting during standing balance. They also reported moderate positive (r = .65) correlations between radial diffusivity in the same region and proprioceptive reweighting [73]. These findings, in the same directions as ours, reveal a consistent pattern in microstructural changes associated with trunk control in cerebellar and now cortical sensorimotor regions.

In addition to white matter microstructure, we also assessed pairwise anatomical connectivity between cortical sensorimotor regions of interest. We limited our regions of interest to cortical sensorimotor areas because our hypothesis was that impairments in cortical sensorimotor integration drive the altered trunk control seen in cLBP. Further, this network has limited exploration in the literature, and no previous diffusion MRI study in cLBP has specifically addressed these regions. We did not find group differences between cLBP and HC for any region pair. Mao et al. [71] assessed thalamo-cortical anatomical connectivity between individuals with cLBP and HC and reported a group difference in anatomical connectivity between the thalamus and the LM1. Pijnenburg et al. [72] reported a greater nodal degree of LM1 in persons with cLBP using graph theoretical techniques on a network consisting of the 116 regions of the AAL atlas. Considering this evidence, future studies investigating group differences in sensorimotor anatomical connectivity in cLBP should consider including additional cortical and subcortical regions of interest.

In the cLBP group, greater anatomical connectivity between right-hemisphere sensory (S1, PO) and left-hemisphere motor (M1, SMC) regions was moderately associated with better trunk control. These findings provide evidence for the importance of interhemispheric sensorimotor information exchange for trunk control. Proprioceptive information from the left side of the body is encoded in RS1 [1]. The PO integrates multi-modal sensory information for movement control and contributes to the brain’s ability to plan and modify movements [115–118]. Greater anatomical connectivity between right-hemisphere sensory and left-hemisphere motor areas may represent larger white matter “highways,” allowing for information exchange between the two sides of the trunk needed for more effective movement planning (SMC) and execution (M1). This may be an adaptation to pain or musculoskeletal impairments. Our findings align with studies in older adults and persons with multiple sclerosis, which have consistently reported relationships between WM structure in the corpus callosum and trunk control performance in the same direction [119–121]. We found positive relationships between SPL-SMC anatomical connectivity and trunk control in both conditions, where greater connectivity between these areas was associated with worse task performance. The SPL is a major component of a distributed spatial attention network, and its function has been related to the modification of spatial reference frames linked to attentional priorities [122–124]. Our trunk control task requires attending to intra- and extra-personal stimuli with continuous updating of spatial reference frames. In the cLBP group, greater anatomical connectivity involving the SPL in those who performed worse on the task could be compensation for chronic pain and disrupted proprioception [125]. Findings implicating right-sided sensory regions in this study also align with previous work suggesting sensory regions in the right hemisphere are asymmetrically involved in online monitoring of trunk control [4].

A greater number of significant relationships between white matter microstructure, anatomical connectivity, and trunk control support that individuals with cLBP adopt a more cortically driven sensorimotor integration strategy for this task compared to HC. If cortical sensorimotor network structure and function were the same in both groups, we would assume that the direction and strength of observed brain/behavior relationships would be the same. Our findings suggest this is not the case. Given the differences in trunk control between groups, a cortically driven strategy may be suboptimal. Cortical involvement in trunk control has been shown to increase with aging [16, 17] and cortically driven sensorimotor integration strategies have been described in individuals with persistent functional limitations following anterior cruciate ligament injury in different motor control tasks [18, 19]. To further tease out the relative contributions of cortical and subcortical regions, future research should consider brain models that include subcortical sensorimotor and cerebellar regions. Differences in connectivity between subcortical and cerebellar regions and their connections to the cortical sensorimotor network may exist between groups and provide context for the relationships observed between anatomical connectivity and trunk control in this study.

While our study design does not allow us to determine causality, evidence of a more cortically driven trunk control strategy in the cLBP group suggests that treatments for impaired trunk control in this population should target more efficient cortical sensorimotor integration. This may occur through restoring normal somatosensation by reducing pain and restoring accurate proprioception. Compared with subcortical brain activity, cortical brain activity is associated with more conscious and voluntary movement. Therefore, individuals with cLBP may benefit from motor control training that adopts implicit motor learning strategies and reduces the attention given to specific body regions and positions. Addressing maladaptive thoughts, attitudes, and beliefs related to kinesiophobia and pain catastrophizing may also reduce attentional focus on the trunk. Additionally, combining non-invasive brain stimulation, including transcranial magnetic stimulation (TMS) and transcranial direct current stimulation (tDCS), with specific trunk control training may enhance rehabilitation outcomes.

This study has several limitations. We did not perform an a priori power analysis because this data was collected as part of a larger study seeking to answer a related but different question. Additionally, our sample size was relatively small (n = 67), especially considering the high dimensionality of brain imaging data. These two factors increase the likelihood of type II error and may have influenced our ability to detect between-group differences in white matter microstructure and anatomical connectivity.

Another limitation of this study was the heterogeneity in the presence or absence of trunk control impairment in the cLBP group, meaning some in this group demonstrated trunk control similar to that of HC. This is partially exhibited by the bimodal distribution of seated trunk control task performance and larger between-subject variability in cLBP compared with HC (Fig 4). Subgroups of individuals with and without motor control impairment have been reported previously in the cLBP literature [126, 127]. We believe this heterogeneity, along with the moderate pain and low level of disability in our cLBP sample, may have impacted our ability to detect between-group differences for measures of cortical sensorimotor white matter. We would not necessarily expect to find cortical sensorimotor WM changes in the absence of impaired sensorimotor control, or at least not to the same extent. A greater range of scores across subjects for task performance in the cLBP group may also contribute to stronger correlations between measures of cortical sensorimotor matter white matter and trunk control. It is possible that for the HC group, the current trunk control task was not challenging enough to provide the spread of scores needed to detect associations.

We implemented two diffusion MRI techniques that model the same diffusion data in different ways, both with limitations. The data presented on microstructure utilizes a tensor model [104]. This approach has been widely adopted and is sensitive to detect differences in clinical populations and demonstrate relationships between brain and behavior [128]. Assessing mean fractional anisotropy in the entire cortical sensorimotor network may have influenced our sensitivity to detect effects. We may have found different results if we considered the tract bundles that make up this network separately. This could help with the known limitations of the tensor model in areas with crossing fibers. It would also narrow in on target brain areas for prevention and intervention strategies. Conversely, the data we present on region-to-region anatomical connectivity uses a ball-and-stick model that allows for modeling multiple fiber directions in a single voxel [108], which is particularly useful for brain areas that involve multiple fiber bundles with different directions [105]. Probabilistic tractography also has limitations, including being an indirect measure and the risk of generating false positives and negatives in areas of low signal or complex fiber architecture [129, 130]. While both approaches have strengths and limitations; we believe the combination of methods to be complementary.

## 5 Conclusion

Persons with cLBP demonstrate worse trunk control performance compared with HC. Although no differences in cortical sensorimotor white matter were observed between groups, cortical sensorimotor microstructure and anatomical connectivity were related to trunk control performance in the cLBP group. The relationships between white matter microstructure and trunk control in cLBP suggest that impairments in trunk control may be related to changes in myelination within cortical sensorimotor pathways, as opposed to axonal degeneration, injury, or loss. For persons with cLBP, anatomical connectivity between ROIs suggests that better trunk control was associated with dynamic integration of interhemispheric information. Poorer trunk control may be related to motor planning strategies that rely on attentionally driven changes in spatial reference frames. Fewer and weaker relationships were observed between white matter measures and task performance in the HC group, suggesting that individuals with cLBP may adopt a more cortically driven sensory integration strategy to meet trunk control task demands. Future research should strive to replicate these findings, with the addition of subcortical and cerebellar structures, and identify therapeutic approaches that could effectively modulate potentially suboptimal movement control strategies.

## Supporting information

S1 Appendix Detailed inclusion and exclusion criteria. (DOCX)

S1 Table Group means, standard deviations, and comparisons for white matter microstructure. (DOCX)

S2 Table Group means, standard deviations, and comparisons for anatomical connectivity. (DOCX)

## List of abbreviations

cLBP: chronic low back pain
HC: healthy control
dMRI: diffusion magnetic resonance imaging
WM: white matter
CEA_95_: 95% confidence ellipse area
M1: primary motor cortex
S1: primary somatosensory cortex
FA: fractional anisotropy
COP: center of pressure
EO: eyes open
EC: eyes closed
TR: response time
TE: echo time
ms: millisecond
mm: millimeter
AAL: Automated Anatomical Labeling
HMAT: human motor area template
SMC: supplementary motor cortex
PO: parietal operculum
FSL: FMRIB software library
ROI: region of interest
MD: mean diffusivity
AD: axial diffusivity
RD: radial diffusivity
