# Supplementary material for: Association between seated trunk control and cortical sensorimotor white matter brain changes in patients with chronic low back pain
Source: PLoS One. 2024 Aug 29;19(8):e0309344. doi: 10.1371/journal.pone.0309344 (PMC11361694; doi:10.1371/journal.pone.0309344)
Supplement: S1 Appendix — (DOCX) [file pone.0309344.s001.docx]

**S1 Appendix. Detailed Inclusion and Exclusion Criteria**.

**Study Inclusion Criteria**

This investigation targeted adults between the ages of 18-65 years old, fluent in English-language (self-report measures in English). This age range ensured skeletal maturity with a reasonably low prevalence of severe osteoarthritis or osteopenia.

**Chronic Low Back Pain Group:** Inclusion criteria were consistent with the Chronic Low Back Pain Minimal Dataset and include: (1) **duration of the current episode of low back symptoms greater than 3 months;** (2) back pain impacting function at least ½ days in last 3-6 months, and (3) ability to identify activities that they were currently unable to perform without pain or limitation.

**Study Exclusion Criteria**

The presence of any of the following factors excluded all participants from this study:

Additional exclusion criteria included the presence of any of the following conditions as determined by prior medical and/or radiographic examination or initial MRI:

- spinal or hip osteoporosis;
- inflammatory joint disease;
- any current (within 5 years) neoplastic condition;
- any history of a vertebral fracture with current bony instability or measurable deformity;
- severe lumbar stenosis (defined as an A-P diameter of the thecal sac of less than 5 mm at any level, from mid-sagittal lumbar T_2_-weighted MRI);
- any abnormalities or compression of the spinal cord or cauda equina;
- compression of a spinal nerve with accompanying clinical symptoms that demonstrate significant loss of or absence of sensation or muscle weakness;
- any lower extremity peripheral nerve impairment;
- injury to your legs that significantly limits your hip or knee motion;
- unstable angina, congestive heart failure, orthopnea, or severe hypertension;
- any history of a surgical procedure to the lumbar spine;
- any surgical procedures to the abdomen, thorax, upper extremities, head or neck in the 6 months prior to enrollment in the study;
- current use of any of the following medications: prescribed anticoagulants (this does not include low doses of ASA or NSAIDs), and oral or injected corticosteroids;
- current use of narcotic medication (opioids) for your back pain that the patient is unable or unwilling to abstain from for 48 hours,
- severe obesity precluding the participant from fitting in the 24” bore of the MRI scanner. The usual weight limit is approximately 350 lbs.

**Additional Exclusion Criteria**

1). Currently involved in an unsettled worker’s compensation claim or in personal injury litigation.

2). Currently applying for, permanent or temporary disability due to a medical or mental health condition, unsettled case.

3). Activity-limiting pain arising from any site other than listed in the specific entry criteria,

4). The presence of any conditions that would contraindicate lumbar MRI

**Healthy Control Group:** The individuals with this group were age (+/- 5 years) BMI (+/- 3 kg/m^2^) and sex-matched to the cLBP group. They must have self-reported no low back pain for which they have sought medical attention or resulted in greater than 3 days of significant loss of ability to accomplish daily function within the last 5 years.
