# Supplementary material for: Association between seated trunk control and cortical sensorimotor white matter brain changes in patients with chronic low back pain
Source: PLoS One. 2024 Aug 29;19(8):e0309344. doi: 10.1371/journal.pone.0309344 (PMC11361694; doi:10.1371/journal.pone.0309344)
Supplement: S1 Table — (DOCX) [file pone.0309344.s002.docx]

**S2 Table**. **Group Means, Standard Deviations, and Comparisons for White Matter Microstructure**.

| **Table.** Group means, standard deviations, and comparisons for WM microstructure. | | | | | |
| --- | --- | --- | --- | --- | --- |
|  | cLBP | | HC | |  |
| WM Microstructure | Mean | SD | Mean | SD | p-value |
| Tract Volume (mm^3^) | 47468.454 | 13011.155 | 45301.462 | 7775.116 | 0.821 |
| FA | 0.556 | 0.028 | 0.559 | 0.023 | 0.644 |
| MD | 0.000586 | 0.000021 | 0.000588 | 0.000017 | 0.603 |
| RD | 0.000379 | 0.000025 | 0.000379 | 0.000021 | 0.901 |
| AD | 0.000999 | 0.000035 | 0.001007 | 0.000024 | 0.246 |
| *p-value associated with T-test or Mann-Whitney U test for group differences | | | | | |
