# Supplementary material for: Association between seated trunk control and cortical sensorimotor white matter brain changes in patients with chronic low back pain
Source: PLoS One. 2024 Aug 29;19(8):e0309344. doi: 10.1371/journal.pone.0309344 (PMC11361694; doi:10.1371/journal.pone.0309344)
Supplement: S2 Table — (DOCX) [file pone.0309344.s003.docx]

**S3 Table. Group Means, Standard Deviations, and Comparisons for Anatomical Connectivity**.

| **Table**. Group means, standard deviations, and comparisons for anatomical connectivity | | | | | |
| --- | --- | --- | --- | --- | --- |
|  | cLBP | | HC | |  |
| Pairwise Anatomical Connectivity | Mean | SD | Mean | SD | p-value |
| **rm1-rs1** | 4.797 | 0.742 | 4.725 | 0.429 | 0.625 |
| **rm1-rpo** | 9.900 | 3.273 | 10.543 | 3.265 | 0.424 |
| **rm1-rsmc** | 5.418 | 0.748 | 5.538 | 0.743 | 0.512 |
| **rm1-rspl** | 2.375 | 1.127 | 2.437 | 1.294 | 0.861 |
| **rm1-lm1** | 15.094 | 4.688 | 15.518 | 4.026 | 0.692 |
| **rm1-ls1** | 6.336 | 2.995 | 7.118 | 3.089 | 0.298 |
| **rm1-lpo** | 4.681 | 3.320 | 4.938 | 3.285 | 0.841 |
| **rm1-lsmc** | 5.951 | 2.613 | 6.267 | 2.025 | 0.581 |
| **rm1-lspl** | 1.110 | 0.516 | 1.190 | 0.535 | 0.581 |
| **rs1-rpo** | 15.082 | 4.253 | 14.925 | 3.080 | 0.863 |
| **rs1-rsmc** | 2.745 | 0.671 | 2.629 | 0.808 | 0.527 |
| **rs1-rspl** | 5.878 | 0.941 | 5.883 | 0.896 | 0.981 |
| **rs1-lm1** | 3.495 | 1.417 | 3.731 | 2.035 | 0.802 |
| **rs1-ls1** | 11.117 | 3.354 | 11.893 | 3.011 | 0.322 |
| **rs1-lpo** | 7.031 | 4.017 | 7.514 | 4.169 | 0.744 |
| **rs1-lsmc** | 1.203 | 0.312 | 1.238 | 0.321 | 0.655 |
| **rs1-lspl** | 7.369 | 3.332 | 7.956 | 4.242 | 0.851 |
| **rpo-rsmc** | 5.639 | 2.252 | 5.985 | 2.593 | 0.802 |
| **rpo-rspl** | 6.732 | 3.321 | 6.412 | 3.700 | 0.555 |
| **rpo-lm1** | 4.046 | 2.761 | 3.930 | 1.992 | 0.763 |
| **rpo-ls1** | 9.633 | 4.512 | 9.084 | 3.512 | 0.578 |
| **rpo-lpo** | 2.362 | 2.814 | 1.798 | 1.583 | 0.522 |
| **rpo-lsmc** | 2.221 | 1.452 | 2.029 | 0.875 | 0.851 |
| **rpo-lspl** | 3.719 | 2.878 | 3.155 | 2.042 | 0.572 |
| **rsmc-rspl** | 2.735 | 1.349 | 2.965 | 1.635 | 0.535 |
| **rsmc-lm1** | 9.771 | 4.343 | 9.868 | 3.269 | 0.918 |
| **rsmc-ls1** | 1.385 | 0.718 | 1.549 | 0.879 | 0.679 |
| **rsmc-lpo** | 2.561 | 1.232 | 2.485 | 1.338 | 0.564 |
| **rsmc-lsmc** | 25.257 | 5.272 | 25.158 | 4.404 | 0.934 |
| **rsmc-lspl** | 1.274 | 0.475 | 1.228 | 0.351 | 0.642 |
| **rspl-lm1** | 0.793 | 0.418 | 0.906 | 0.562 | 0.474 |
| **rspl-ls1** | 3.147 | 2.442 | 4.039 | 2.861 | 0.160 |
| **rspl-lpo** | 1.395 | 0.969 | 1.872 | 1.436 | 0.043 |
| **rspl-lsmc** | 1.096 | 0.483 | 1.272 | 0.588 | 0.205 |
| **rspl-lspl** | 13.682 | 7.240 | 15.887 | 5.590 | 0.166 |
| **lm1-ls1** | 4.573 | 0.727 | 4.788 | 0.654 | 0.207 |
| **lm1-lpo** | 9.912 | 3.396 | 9.568 | 3.570 | 0.688 |
| **lm1-lsmc** | 5.826 | 0.831 | 6.086 | 0.687 | 0.166 |
| **lm1-lspl** | 3.099 | 1.106 | 3.405 | 1.434 | 0.303 |
| **ls1-lpo** | 12.383 | 3.821 | 12.454 | 3.557 | 0.938 |
| **ls1-lsmc** | 2.672 | 0.874 | 2.707 | 0.959 | 0.514 |
| **ls1-lspl** | 5.802 | 1.085 | 5.767 | 0.769 | 0.878 |
| **lpo-lsmc** | 9.134 | 3.842 | 8.710 | 3.527 | 0.639 |
| **lpo-lspl** | 6.355 | 3.066 | 6.370 | 2.991 | 0.811 |
| **lsmc-lspl** | 3.483 | 1.387 | 3.677 | 1.318 | 0.560 |
| *p-value associated with T-test or Mann-Whitney U test for group differences. | | | | | |
